# Combination of smoking and Epstein-Barr virus DNA is a predictor of poor prognosis for nasopharyngeal carcinoma: a long-term follow-up retrospective study

**DOI:** 10.1186/s12885-022-10297-w

**Published:** 2022-12-05

**Authors:** Wanxia Li, Chao Yang, Feipeng Zhao, Junzheng Li, Zonghua Li, Ping Ouyang, Xiaofei Yuan, Shuting Wu, Yue Yuan, Linchong Cui, Huiru Feng, Danfan Lin, Zilu Chen, Juan Lu, Xiaohong Peng, Jing Chen

**Affiliations:** 1 Department of Otolaryngology-Head and Neck Surgery, Nanfang Hospital, Southern Medical University, Guangzhou, 510515 Guangdong China; 2 Department of Health Management, Nanfang Hospital, Southern Medical University, Guangzhou, 510515 Guangdong China; 3 Department of Laboratory Medicine, Nanfang Hospital, Southern Medical University, Guangzhou, 510515 Guangdong China; 4 Department of Otolaryngology-Head and Neck Surgery, Chengdu Second People’s Hospital, Chengdu, 610000 Sichuan China; 5 Department of Otolaryngology-Head and Neck Surgery, Zhujiang Hospital, Southern Medical University, Guangzhou, 510220 Guangdong China; 6 Department of Otolaryngology, 942 Hospital of the Chinese People’s Liberation Army, Yinchuan, 750001 Ningxia China; 7 School of Traditional Chinese Medicine, Southern Medical University, Guangzhou, 510515 Guangdong China

**Keywords:** Smoking, EBV DNA, Prognosis, Combination, Nasopharyngeal carcinoma

## Abstract

**Background:**

This retrospective study was performed to determine the prognostic potential of smoking and its combination with pre-treatment plasma Epstein-Barr virus (EBV) DNA levels in patients with nasopharyngeal carcinoma (NPC).

**Methods:**

Medical records of 1080 non-metastatic NPC patients who received intensity-modulated radiotherapy were reviewed. Male patients were categorized as never and ever smokers, and the smoking amount, duration, and cumulative consumption were used to evaluate dose-dependent effects. Survival outcomes were assessed using Kaplan-Meier survival analysis and the multivariate Cox regression analysis. Propensity score matching (PSM) was constructed.

**Results:**

The 5-year overall survival (OS) was worse for ever smokers than never smokers, and significantly decreased with the increase of smoking amount, duration, and cumulative consumption. Compared with never smokers, the multivariate-adjusted hazard ratio (HR) of death was higher in ever smokers (HR = 1.361, *P* = 0.049), those smoked ≥20 cigarettes/day (HR = 1.473, *P* = 0.017), those smoked for ≥30 years (HR = 1.523, *P* = 0.023), and those cumulative smoked for ≥30 pack-years (HR = 1.649, *P* = 0.005). The poor prognostic effects of smoking was also confirmed in the PSM analysis. The combination of cumulative smoking consumption and pre-treatment EBV DNA levels was proven to be an independent poor prognostic factor for male NPC, and the risk of death, progression, and distant metastases gradually increased with both factors (*P* < 0.001).

**Conclusions:**

Combination of smoking and pre-treatment EBV DNA levels as a predictor of poor prognosis could further improve the risk stratification and prognostication for NPC.

**Supplementary Information:**

The online version contains supplementary material available at 10.1186/s12885-022-10297-w.

## Background

Nasopharyngeal carcinoma (NPC) is one of the most aggressive head and neck tumor, which is especially prevalent in southern China [1]. Multiple risk factors, including host genetics, Epstein–Barr virus (EBV) infection, and environmental factors, had been confirmed to contribute to the development of NPC [2]. Tobacco is classified as a group 1 carcinogen by the International Agency for Research on Cancer (IARC) [3], and has proven to be a significant predictor of a poor prognosis for patients with a wide variety of malignancies [4, 5], including head and neck tumors [6, 7] such as oropharyngeal and laryngeal carcinomas [8, 9].

As cigarette smoking is a adverse but preventable lifestyle factor contributing to global cancer deaths [10, 11], its prognostic value for NPC has also recently attracted research attention. In 2012, Shen et al. [12] provided the first prospective evidence that pre-treatment smoking was linked to a worse prognosis of NPC patients. Subsequently, studies conducted by Chen et al. [13] and Lin et al. [14] showed that smokers suffered higher risk of death than non-smokers, and the risk increased in a dose-response relation with the daily number of cigarettes smoking and cumulative consumption. Moreover, two other retrospective studies suggested that smoking was associated with the overall survival (OS), locoregional relapse-free survival (LRFS), distant metastasis-free survival (DMFS), and progression-free survival (PFS) of NPC patients [15, 16]. However, Guo et al. [17] did not find a correlation between ever smoked and OS, distant failure-free survival, and failure-free survival in locoregionally advanced NPC patients. Although these previous studies have suggested that smoking is a potential prognostic risk factor with a dose-dependent effect for NPC patients, these studies suffered from limitations of relatively small sample sizes, short follow-up duration, a single endpoint, or inconsistent results. Therefore, further exploration is needed.

It is well known that EBV reactivation plays a causal role in the development of NPC [18]. Interestingly, smoking was reported to be associated with EBV seropositivity, and cigarette smoke extracts can promote EBV latent-to-lytic activation in vitro [19]. A recent prospective study supported this conclusion, which showed that cigarette smoking is likely to notably increase the risk of developing NPC partly by repeatedly reactivating EBV [20]. Since smoking is associated with EBV reactivation and that plasma EBV DNA is a strong independent prognostic factor for NPC [21], smoking and EBV DNA may have joint effects on the prognosis of NPC patients. To date, only one retrospective study has suggested that a comprehensive evaluation of smoking and baseline EBV DNA was an independent prognostic factor. This finding refines the risk stratification for NPC patients undergoing intensity-modulated radiotherapy (IMRT), especially those with high baseline EBV DNA load [22]. Therefore, the potential prognostic value of the combination of smoking and pre-treatment EBV DNA levels for NPC is far from conclusive and warrants further investigation.

We performed a long-term follow-up retrospective study to explore the prognostic impact of cigarette smoking in NPC patients and to assess whether combining smoking and the pre-treatment plasma EBV DNA levels has further prognostic value for NPC.

## Methods

### Patients

The medical records of 1748 primary diagnosed with biopsy-proven NPC patients in our institution from March 2005 to December 2015 were reviewed. The inclusion criteria were as follows: (1) histologically confirmed non-keratinized NPC; (2) primary diagnosed patients without evidence of systemic metastasis; (3) availability of complete clinical information including smoking history; (4) completion of scheduled therapy; and (5) no history of malignancy or concurrent cancer. The exclusion criteria included: (1) lack of complete medical information, including smoking history data; (2) distant metastasis had been confirmed at initial diagnosis; (3) failure to complete treatment or death during treatment; and (4) prior or other concurrent malignancies. Records of 1080 patients in total were eventually enrolled in this research. Patients were staged in terms of the 7th edition of the AJCC clinical stage system [23]. Detailed information regarding the treatment and follow-up regimens for the whole cohort is illustrated in the Supplementary Material 1–2.

### Cigarette smoking assessment

Information on smoking habits included smoking status (ever smokers or never smokers), smoking amount (number of cigarettes smoked per day), smoking duration (years smoked), and cumulative smoking consumption. Ever smokers included both current and former smokers, defined as patients who smoked within the last year or who had quit smoking for for at least 1 year, respectively. Given that the number of former smokers (20 patients) was too small for statistical analysis, we combined current and former smokers into a single group of ever smokers. In addition to the contribution of overall smoking status (never-smokers vs. ever-smokers), we further assessed the smoking amount (0, 1–19, ≥20 cigarettes/day), smoking duration (0, 1–29, ≥30 years), and cumulative smoking consumption (0, 1–29, ≥30 pack-years) according to previous studies [13–17]. Cumulative smoking consumption was calculated as pack-years, pack-years = [smoking amount (cigarettes/day) × smoking duration (years smoked)] /20(20 cigarettes per pack).

### Measurement of plasma EBV DNA levels

Measurements of plasma EBV DNA were detected by a real-time quantitative polymerase chain reaction (PCR) before treatment, using the same method and procedure described in our previous publications [24, 25]. All plasma EBV DNA tests were performed at the department of Laboratory Medicine of our hospital. 0 copies/mL was defined as a negative result for plasma EBV DNA load and > 0 copies/mL was considered as a positive plasma EBV DNA level. In this present study, there were 673 male patients with available pre-treatment plasma EBV DNA test records. Referring to our previous researches [24, 25], the cutoff value for pre-treatment EBV DNA used to classify patients into low and high groups was 1500 copies/mL.

### Endpoints and statistical analysis

The primary study endpoint was OS, and the secondary endpoints included PFS, DMFS, and LRFS as defined previously [24, 25] and detailed definition can also be found in Supplementary Material 3. SPSS software version 25.0 was used for performing statistical and PSM analysis. The chi-square test or Fisher’s exact test was performed to compare the differences in patients’ baseline characteristics according to smoking status. Survival comparisons between groups were estimated using Kaplan-Meier survival curves and log-rank test. Hazard ratios (HR) and 95% confidence intervals (CI) were calculated in multivariate Cox regression analysis. Ever smokers group were matched with never smokers group by 1:1 PSM with a caliper value of 0.05; matching factors included age, overall stage, tumor stage, node stage, alcohol drinking, and family history of NPC (Supplementary Material 5). A two-tailed *P* value of less than 0.05 were deemed statistically significant.

## Results

### Demographic and smoking characteristics

Baseline characteristics of the 1080 patients with a median age of 46.2 years (range: 12–80 years) are listed in Table 1. Considering that the number of female smokers (4 patients) was too small, we considered only the male patients (*N* = 793) in the statistical analysis to eliminate the confounding effect of gender. The characteristics of the 793 male patients and 566 PSM matched male patients grouped in term of smoking status are shown in Table 1 and Supplementary Material 5, and the proportions according to all smoking indicators are seen in Supplementary Table 1. The median follow-up time was 66.4 months (range: 2–197 months) and the 5-year OS, PFS, LRFS, and DMFS rates were 79.5, 64.6, 87.1, and 78.2%, respectively. Detailed information for treatment failure is presented in Supplementary Material 4.

**Table 1:** Clinical characteristics of patients with NPC

Characteristic	Total patients (*N* = 1080)			Male patients (*N* = 793)			PSM matched male patients (*N* = 566)		
	Never smokers (*N* = 621) n (%)	Ever smokers (*N* = 459) n (%)	*P* value^*^	Never smokers (*N* = 338) n (%)	Ever smokers (*N* = 455) n (%)	*P* value^*^	Never smokers (*N* = 283) n (%)	Ever smokers (*N* = 283) n (%)	*P* value^#^
**Sex**			< 0.001			–			–
**Female**	283 (45.6%)	4 (0.9%)		0 (0)	0 (0)		0 (0)	0 (0)	
**Male**	338 (54.4%)	455 (99.1%)		338 (100%)	455 (100%)		283 (100%)	283 (100%)	
**Age (years)**			< 0.001			< 0.001			1.000
**< 45**	321 (50.2%)	161 (35.1%)		177 (52.4%)	160 (35.2%)		133 (47.0%)	132 (46.6%)	
**≥ 45**	309 (49.8%)	298 (64.9%)		161 (47.6%)	295 (64.8%)		150 (53.0%)	151 (53.4%)	
**Overall stage**^**b**^			0.050			0.051			0.706
**I**	48 (7.7%)	28 (6.1%)		26 (7.7%)	28 (6.2%)		25 (8.8%)	22 (7.8%)	
**II**	90 (14.5%)	55 (12.0%)		55 (16.3%)	55 (12.1%)		40 (14.1%)	39 (13.8%)	
**III**	211 (34.0%)	133 (29.6%)		112 (33.1%)	133 (29.2%)		86 (30.4%)	86 (30.4%)	
**IV**	272 (43.8%)	239 (52.3%)		145 (42.9%)	239 (52.5%)		132 (46.6%)	136 (48.1%)	
**Tumor stage**^**b**^			0.059			0.044			0.085
**T1**	129 (20.8%)	93 (20.3%)		70 (20.7%)	93 (20.4%)		62 (21.9%)	66 (23.3%)	
**T2**	127 (20.5%)	86 (18.7%)		70 (20.7%)	84 (18.5%)		57 (20.1%)	57 (20.1%)	
**T3**	133 (21.4%)	75 (16.3%)		76 (22.5%)	74 (16.3%)		52 (18.4%)	41 (14.5%)	
**T4**	232 (37.4%)	205 (44.7%)		122 (36.1%)	204 (44.8%)		112 (39.6%)	119 (42.0%)	
**Node stage**^**b**^			0.295			0.052			0.192
**N0**	97 (17.8%)	64 (13.9%)		60 (17.8%)	64 (14.1%)		50 (17.7%)	50 (17.7%)	
**N1**	188 (32.8%)	125 (27.2%)		111 (32.8%)	125 (27.5%)		84 (29.7%)	93 (32.9%)	
**N2**	290 (42.9%)	224 (48.8%)		145 (42.9%)	220 (44.8%)		130 (45.9%)	120 (42.4%)	
**N3**	46 (6.5%)	46 (10.1%)		22 (6.5%)	46 (10.1%)		19 (6.7%)	20 (7.1%)	
**Alcohol drinking**			< 0.001			< 0.001			1.000
**No**	605 (97.4%)	328 (71.5%)		322 (95.3%)	324 (71.2%)		267 (94.3%)	267 (94.3%)	
**Yes**	16 (2.6%)	131 (28.5%)		16 (4.7%)	131 (28.8%)		16 (5.7%)	16 (5.7%)	
**Family history of NPC**			0.135			0.047			1.000
**No**	576 (92.8%)	436 (95.0%)		309 (91.4%)	432 (94.9%)		265 (93.6)	264 (93.3%)	
**Yes**	45 (7.2%)	23 (5.0%)		29 (8.6%)	23 (5.1%)		18 (6.4%)	19 (6.7%)	

^a^ Pathologic type according to the 2005 World Health Organization (WHO) classification of tumors

^b^ According to the 7th edition of the AJCC staging system

*Chi-square test or Fisher’s exact test

^#^Cochran-Mantel-Haenszel chi-squared test

### Impact of cigarette smoking on survival outcomes

**Fig. 1:**
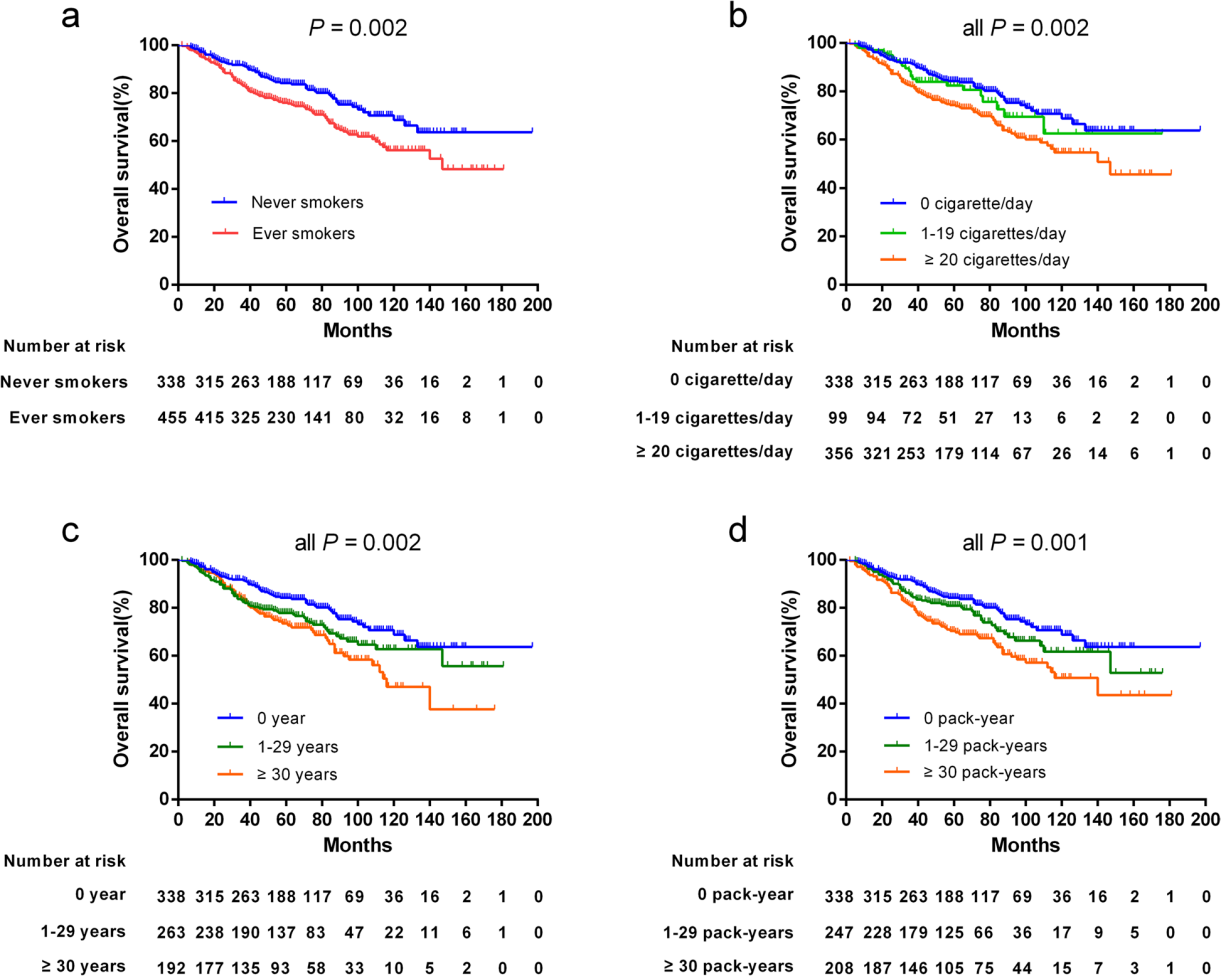
Kaplan-Meier curves for overall survival of male patients stratified by different smoking indicators: **a** smoking status. **b** smoking amount. **c** smoking duration. **d** cumulative smoking consumption

**Fig. 2:**
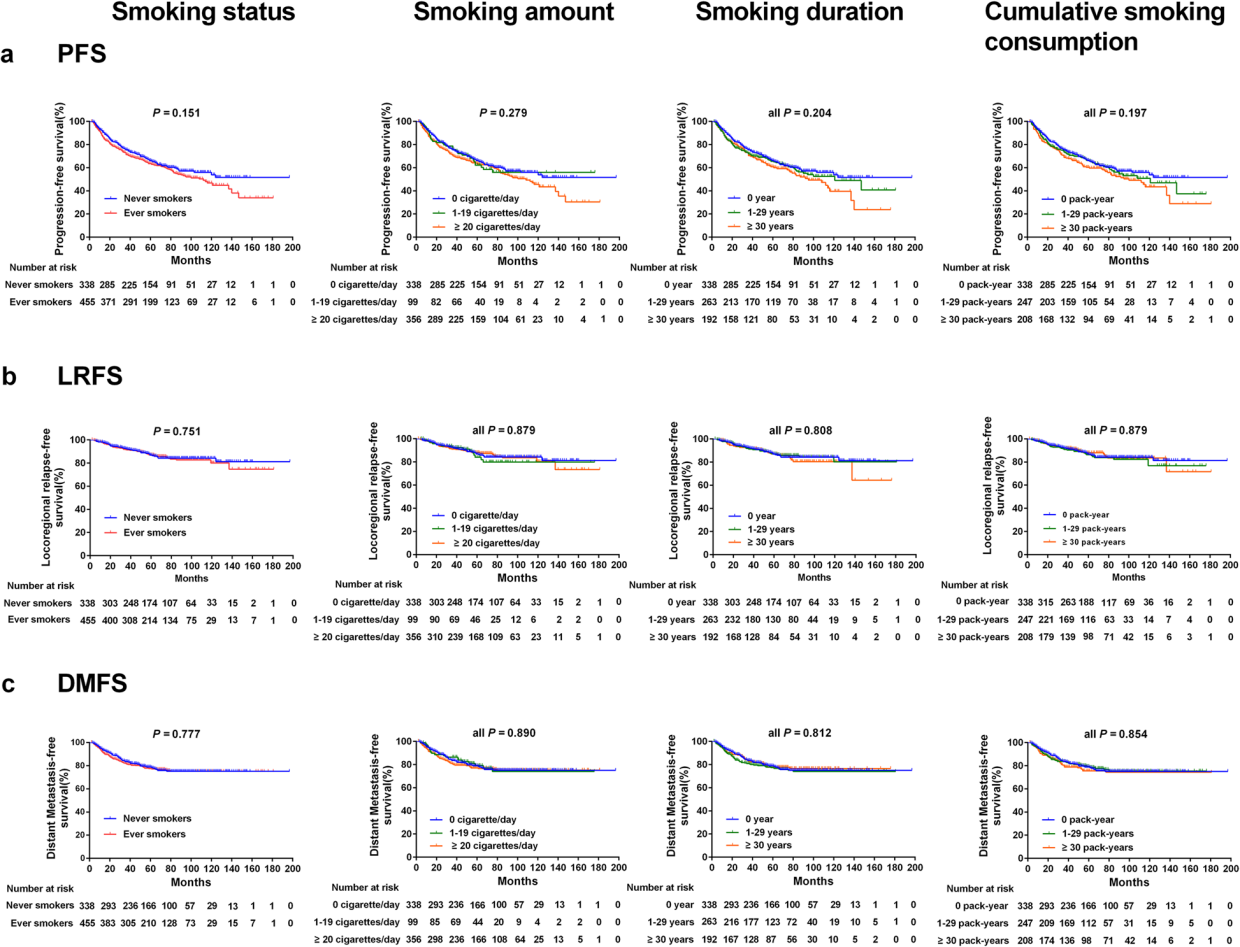
Kaplan-Meier curves for the secondary endpoint survival outcomes of male patients stratified by different smoking indicators. **a** Progression-free survival. **b** Locoregional relapse-free survival. **c** Distant metastasis-free survival

Among male patients, ever smokers had a worse 5-year OS than never smokers (*P* = 0.002), similar significant differences were observed in the categories of smoking amount (*P* = 0.002), smoking duration (*P* = 0.002), and cumulative smoking consumption (*P* = 0.001) (Fig. 1), the above results were also confirmed in the PSM analysis (Supplementary Material 5). However, there were no significant associations with these indices in terms of PFS, LRFS, and DMFS (all *P* > 0.05) (Fig. 2).

### Independent prognostic impact of cigarette smoking based on multivariate analysis

**Fig. 3:**
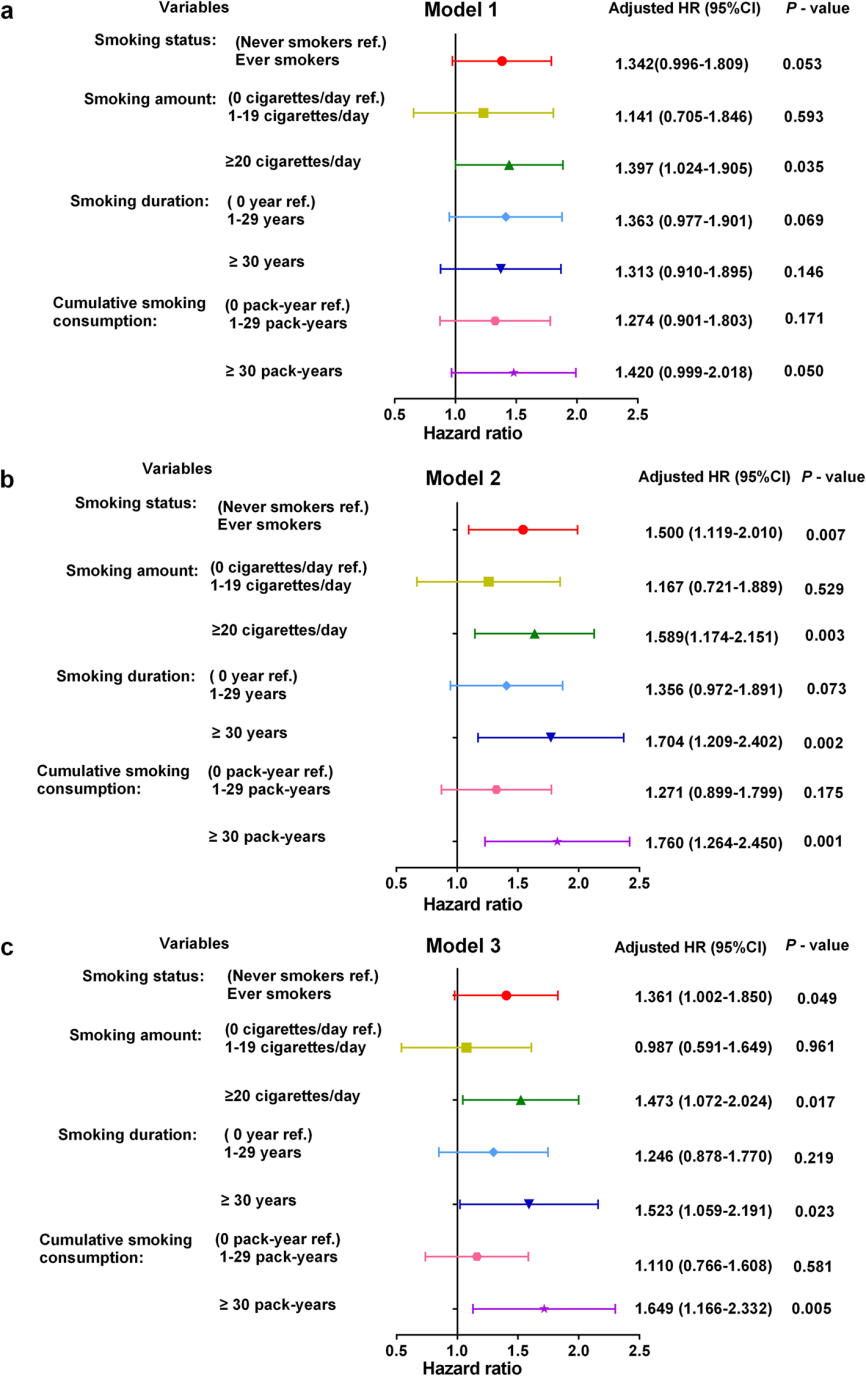
Forest plots of the prognostic effects for smoking indicators on overall survival in male patients. **a** Model 1, performed in the entire cohort, adjusted for age, T stage, and N stage. **b** Model 2, performed in the entire cohort, excluding age as a covariate from Model 1. **c** Model 3, adjusted for T stage and N stage and pre-treatment EBV DNA, performed in 673 patients with these data available

Model 1 of the Cox proportional hazard regression model included the variables age (< 45 vs. ≥45 years), T stage (T1–3 vs. T4), N stage (N0–1 vs. N2–3), and smoking indicators. Only smoking ≥20 cigarettes/day was significantly associated with OS, whereas being an ever smoker and ≥ 30 pack-years of cumulative consumption showed marginal significant effects on a worse OS compared with that of never smokers (Fig. 3a). Considering that age was an imbalanced covariate (Table 1), which was associated with smoking duration and cumulative smoking consumption [26] and therefore might have a confounding effect, Model 2 excluded age as a covariate. As shown in Model 2, ever smokers, those who smoked ≥20 cigarettes/day, ≥30 years, and ≥ 30 pack-years had a significantly higher risk of death compared with never-smokers (Fig. 3b).

Since a high EBV DNA levels have been identified as a risk factor for unfavourable prognosis in NPC (Supplementary Table 2), patients who had available data of pre-treatment plasma EBV DNA were also included in Model 3. After adjusting for the pre-treatment EBV DNA levels (≥1500 vs. < 1500 copies/mL), ever smoking, smoking ≥20 cigarettes/day, smoking ≥30 years, and cumulative smoking ≥30 pack-years remained independent risk factors for OS of male patients (Fig. 3c). Therefore, smoking was an independent prognostic factor for OS of male NPC patients, and the risk of death increased with smoking amount, smoking duration, and cumulative smoking consumption.

### Combined prognostic value of smoking and pre-treatment EBV DNA

To assess the joint effects of smoking and pre-treatment EBV DNA levels for NPC prognosis, we stratified the 673 male patients with available pre-treatment EBV DNA data into the following four subgroups: (1) cumulative consumption < 30 pack-years and pre-treatment EBV DNA < 1500 copies/mL (*n* = 297); (2) cumulative consumption ≥30 pack-years and pre-treatment EBV DNA < 1500 copies/mL (*n* = 97); (3) cumulative consumption < 30 pack-years and pre-treatment EBV DNA ≥ 1500 copies/mL (*n* = 201); and (4) cumulative consumption ≥30 pack-years and pre-treatment EBV DNA ≥1500 copies/mL (*n* = 78).

**Fig. 4:**
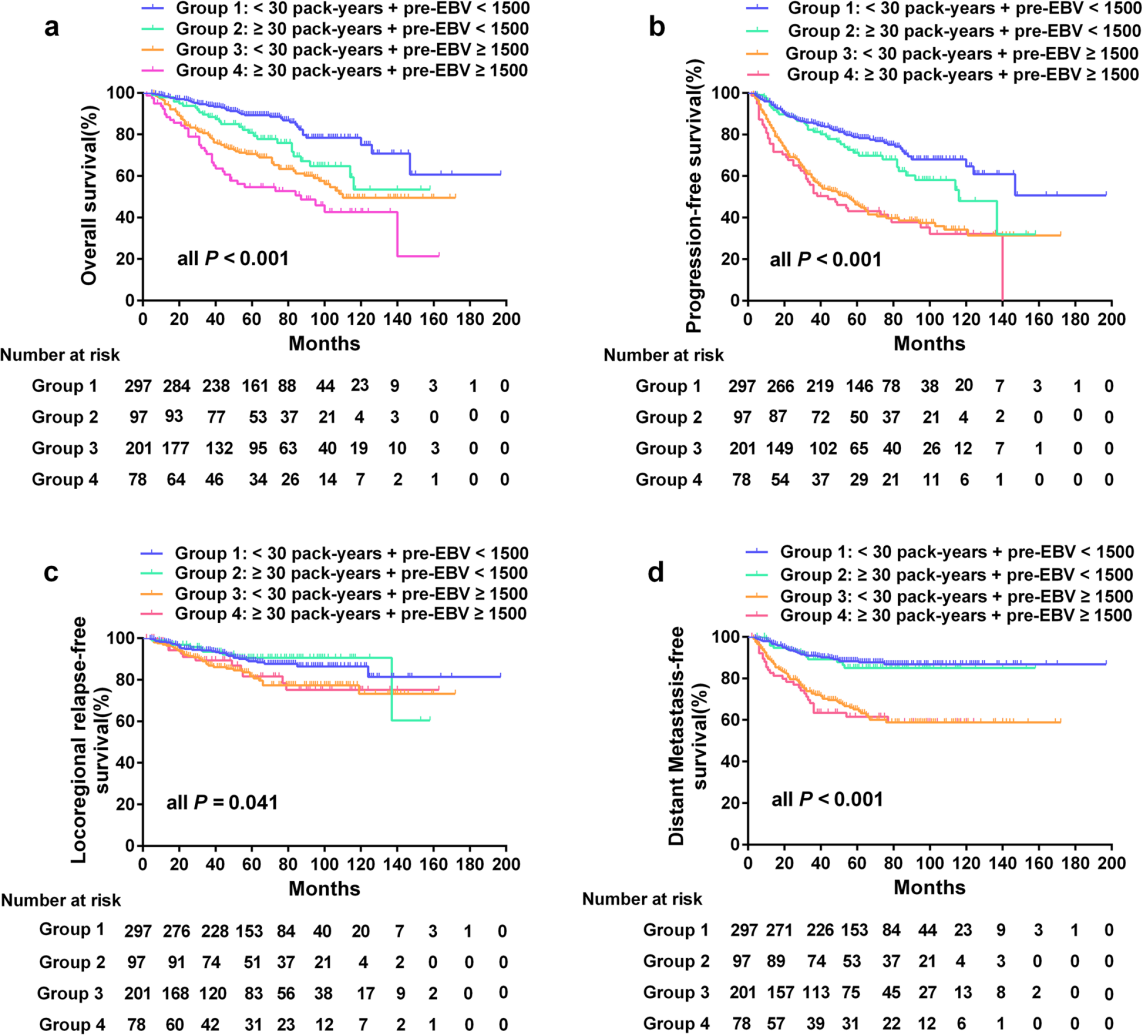
Kaplan-Meier curves for survival outcomes of 673 male patients stratified by cumulative smoking consumption and pre-treatment EBV DNA levels. **a** Overall survival. **b** Progression-free survival. **c** Locoregional relapse-free survival. **d** Distant metastasis-free survival

Differences for the 5-year OS among the above four subgroups were significant (*P* < 0.001), with a clear decreasing trend from group 1 to group 4 (Fig. 4a). Although significant differences also existed in 5-year PFS, LRFS, and DMFS among the four subgroups, these differences were mainly caused by the pre-treatment EBV DNA levels grouping based on the Kaplan-Meier survival curves (Fig. 4b–d).

We further performed a multivariate analysis adjusted for T stage and N stage. The combined stratification of smoking and pre-treatment EBV DNA emerged as an independent prognostic factor for OS with a gradual increase in the HR from Group 2 to Group 4 (all *P* < 0.05). In the low pre-treatment EBV group, patients with a cumulative consumption ≥30 pack-years had a higher risk of death compared with that of patients with cumulative consumption of < 30 pack-years (Table 2). A similar trend was found in the high pre-treatment EBV group, the risk of death for patients with a cumulative consumption of < 30 pack-years was lower than that of the patients with consumption of ≥30 pack-years. A higher HR for patients with a cumulative consumption ≥30 pack-years was also found in the high pre-EBV group for PFS and DMFS, but not for LRFS (Table 2).

**Table 2:** Multivariable Cox proportional hazards models for the combination of cumulative smoking consumption and pre-treatment EBV DNA

Variable	OS^a^		PFS^b^		LRFS^c^		DMFS^d^	
	HR^e^ (95%CI)	*P* value	HR (95%CI)	*P* value	HR (95%CI)	*P* value	HR (95%CI)	*P* value
**T stage** (T4 vs. T1–3)	1.365 (1.012–1.840)	0.041	1.309 (1.027–1.668)	0.030	1.883 (1.219–2.907)	0.004	1.218 (0.874–1.696)	0.245
**N stage** (N2–3 vs. N0–1)	1.230 (0.906–1.670)	0.184	1.217 (0.950–1.559)	0.121	0.765 (0.497–1.175)	0.221	1.890 (1.310–2.726)	0.001
**Combination of cumulative smoking consumption and pre-treatment EBV**^**f**^		< 0.001		< 0.001		0.085		< 0.001
**Group 1:** < 30 pack-years and pre-treatment EBV < 1500	Reference		Reference		Reference		Reference	
**Group 2:** ≥30 pack-years and pre-treatment EBV < 1500	1.836 (1.127–2.991)	0.015	1.394 (0.933–2.083)	0.105	0.806 (0.384–1.692)	0.568	1.090 (0.574–2.069)	0.792
**Group 3:** < 30 pack-years and pre-treatment EBV ≥ 1500	2.519 (1.703–3.728)	< 0.001	2.669 (1.974–3.608)	< 0.001	1.700 (1.035–2.793)	0.036	3.055 (2.012–4.639)	< 0.001
**Group 4:** ≥30 pack-years and pre-treatment EBV ≥ 1500	3.614 (2.308–5.658)	< 0.001	2.912 (2.010–4.219)	< 0.001	1.515 (0.770–2.981)	0.229	3.422 (2.058–5.691)	< 0.001

^a^*OS* Overall survival

^b^*PFS* Progression-free survival

^c^*DMFS* Distant metastasis-free survival

^d^*LRFS* Locoregional relapse-free survival

^e^*HR* Hazard ratio

^f^*EBV* Epstein-Barr virus

## Discussion

In this present study, our results demonstrated that smoking was an independent prognostic factor of poor overall survival in male NPC patients, with dose-dependent effects on all smoking exposure indicators, including smoking amount, duration, and cumulative consumption. Moreover, the combination of smoking cumulative consumption and pre-treatment EBV DNA levels was confirmed to be an independent factor contributing to a poor prognosis in male NPC patients.

The 5-year OS for smokers significantly decreased not only in relation to smoking status but also with an increase of smoking amount, duration, and cumulative consumption, these results were also confirmed in the PSM analysis. After adjusting for the prognostic effects of age and clinical stage, the effect of smoking on the OS of male NPC showed marginal differences; similar results for smoking duration were found in Lin et al. [14]. However, significant independent prognostic effects were clearly observed after excluding age and after adjusting for the strong prognostic factors of plasma EBV DNA and clinical stage. The death risk of ever smokers was 1.361-fold greater than that of never smokers, and was increased by 1.473-fold, 1.523-fold, and 1.649-fold for those smoking ≥20 cigarettes/day, for ≥30 years, or ≥ 30 pack-years, respectively. Although Guo et al. [17] failed to find a significant correlation between smoking and OS for locoregionally advanced NPC patients, the negative effect of smoking status on the OS for male NPC in our study is consistent with most previous studies [12–16]. This provides more convincing evidence for the prognostic value of smoking in NPC.

Although partial dose-response effects such as smoking amount and cumulative smoking consumption have been reported by Ouyang et al. [15], Chen et al. [13], and Lin et al. [14]. But a more important contribution of our study than theirs was the finding that all exposure indicators, including smoking amount, duration, and cumulative consumption, had significant dose-response effects on OS. Previous meta-analyses showed that cigarette smoking definitely increases the risk of NPC incidence with a dose-dependent effect [27, 28]; smoking-related genetic susceptibility genes, such as the 15q25.1 lung cancer susceptibility locus, which has been verified to influence the intensity, duration, and cumulative consumption of cigarette exposure, may be associated with this dose-dependent effect [29]. However, the pathophysiological mechanisms for the dose-dependent relationship between cigarette smoking and NPC prognosis remain unknown and require further molecular analyses.

Some possible mechanisms include the effects of smoking on increasing the serum interleukin-6 levels [30], aggravating tissue hypoxia [31], reducing the sensitivity to chemoradiotherapy [32], inducing the overexpression of oncogenes [33], and activating EBV replication [18]. The latter mechanism has received substantial attention in areas of high NPC incidence in recent years [20, 34–36]. Interestingly, we found that the combination of cigarette smoking and pre-treatment EBV DNA levels had superior prognostic value, which supports and verifies this mechanism to some extent. The risk of death, progression, and distant metastases for male patients gradually increased with an increase in cumulative smoking consumption and EBV DNA levels. Patients with cumulative smoking consumption ≥30 pack-years and pre-EBV ≥1500 copies/mL suffered the highest risk of death, progression, and distant metastases. This finding is broadly consistent with Lv et al. [22] which was the first and only other study to assess the combined prognostic value of smoking and baseline EBV DNA. Thus, the combined prognostic value of these two factors was more significant than that of each factor alone, offering improved prognostic risk stratification. This suggests that particular attention should be paid to heavy and long-term smokers with high EBV DNA levels who may require more intensive treatment and closer clinical surveillance.

Given that EBV reactivation appears to play an important role in the development and progression of NPC [19], we speculate that this joint prognosis effect of smoking and EBV DNA involves the dose-dependent smoking-induced EBV reactivation effect. A multicenter cross-sectional study showed a solid dose-response relationship between current smoking and higher oral EBV loads [34], smokers were 1.59-fold more likely to have detectable plasma EBV DNA than non-smokers [37], and smoking was reported to increase the NPC risk by repeatedly reactivating EBV [20] with more than 90% of this effect mediated through anti-EBV-VCA-IgA [35]. These studies further support our hypothesis. Nevertheless, whether smoking affects the prognosis of NPC by directly activating EBV and the specific pathophysiological mechanism are far from clear and warrant further investigation.

Notably, some patients in our cohort with cumulative smoking consumption ≥30 pack-years also had a higher HR for death than those with < 30 pack-years in the low pre-treatment EBV group, in contrast to the findings of Lv et al. [22]. The main cause of this effect remains unclear, although we suspect that smoking may affect survival through other unknown intervening mediators in NPC patients with low EBV DNA levels. Nevertheless, this finding suggests that smoking has the potential to complement and improve the prognostic risk stratification and prediction regardless of EBV DNA levels, which may provide new insight for improving clinical risk stratification and decision-making.

Our study had several advantages. Firstly, our study is the first research, supported by a reliable PSM analysis, to confirm unfavorable prognostic effect of smoking for male NPC patients with dose-dependent effects on all smoking exposure indicators, including smoking amount, duration, and cumulative consumption. Secondly, we further reveal the combined prognostic predictive value of cumulative smoking consumption and pre-treatment EBV DNA, which suggests the potential for this combination to improve the risk stratification for NPC, and provides new clues for uncovering the mechanism underlying dose-dependent smoking-induced EBV reactivation. Thirdly, we used a long-term follow up database, which reduced the sources of bias and confounding factors, providing more reliable and convincing results.

Nevertheless, some limitations also existed in our study. First, due to the retrospective nature, some potential biases were unavoidable. Potential biases mainly included the fact that the patients in our study were from a single center in an endemic area of NPC; the smoking information of patients was obtained from medical records, rather than using standardized questionnaires at enrollment; patient treatment regimens were not able to achieve the same uniform standards as in the prospective study. Second, since metastatic patients were excluded, the effect of smoking on metastatic patients is beyond the scope of this study and requires further investigation. Third, due to the small sample size of female smokers, they were excluded from this study; therefore, it still uncertain whether the same conclusions could be extrapolated to female patients. Lastly, since smoking may reduce survival of NPC patients by causing other possible non-cancer mortality, such as acute cardiocerebrovascular events and respiratory diseases, rather than by inducing EBV reactivation, the joint prognostic impact of smoking and EBV DNA as well as whether smoking is related to EBV reactivation still needs to be further explored. Hence, larger sample, multi-center, and prospective studies are needed to further examined our findings.

## Conclusions

Smoking was an independent prognostic factor for the poor survival of male NPC patients with a significant dose-dependent effect, and its combination with the pre-treatment EBV DNA levels was a better prognostic predictor, which could further improve risk stratification and prognostication for NPC patients.

## Supplementary Information

**Additional file 1: Supplementary Table 1.** Multivariable analyses of prognostic effects for smoking indicators on overall survival in male patients. **Supplementary Table 2.** Multivariable analyses of prognostic effects for smoking indicators on overall survival in male patients (Details of all variables).

**Additional file 2: Supplementary Material 1.** Detailed treatment regimens for the entire cohort. **Supplementary Material 2.** Detailed information of follow-up for the entire cohort. **Supplementary Material 3**. The detailed definition of the study endpoints. **Supplementary Material 4.** Detailed information of treatment failure for 793 male patients. **Supplementary Material 5.** Detailed information and results of propensity score matching (PSM) analysis. **Supplementary Material 6.** The results of survival analysis of only male smokers who were classified as heavy, moderate and low cigarette smoking consumers.

## Data Availability

All data included in this study are available upon request by contact with the corresponding author.

## Abbreviations

EBV: Epstein-Barr virus
NPC: Nasopharyngeal carcinoma
HR: Hazard ratio
OS: Overall survival
LRFS: Locoregional relapse-free survival
DMFS: Distant metastasis-free survival
PFS: Progression-free survival
IARC: International Agency for Research on Cancer
AJCC: American Joint Committee on Cancer
TNM: Tumor-node-metastasis
IMRT: Intensity-modulated radiotherapy
PCR: Quantitative polymerase chain reaction
CI: Confidence intervals
PSM: Propensity score matching
