# Supplementary material for: Combination of smoking and Epstein-Barr virus DNA is a predictor of poor prognosis for nasopharyngeal carcinoma: a long-term follow-up retrospective study
Source: BMC Cancer. 2022 Dec 5;22:1262. doi: 10.1186/s12885-022-10297-w (PMC9720998; doi:10.1186/s12885-022-10297-w)
Supplement: Supplementary file 1 — Additional file 1: Supplementary Table 1. Multivariable analyses of prognostic effects for smoking indicators on overall survival in male patients. Supplementary Table 2. Multivariable analyses of prognostic effects for smoking indicators on overall survival in male patients (Details of all variables). [file 12885_2022_10297_MOESM1_ESM.doc]

Supplementary Table 1. Multivariable analyses of prognostic effects for smoking indicators on overall survival in male patients.

| **Smoking** **indicators** | **N (%)** | **Univariate analysis** | |  | **Multivariate analysis** | | | | | | | |
| --- | --- | --- | --- | --- | --- | --- | --- | --- | --- | --- | --- | --- |
|  |  | **HR (95%CI)** | **P** |  | **Model 1** | |  | **Model 2** | |  | **Model 3** | |
|  |  |  |  |  | **HR (95%CI)** | **P** |  | **HR (95%CI)** | **P** |  | **HR (95%CI)** | **P** |
| **Smoking status** |  |  | 0.002 |  |  | 0.053 |  |  | 0.007 |  |  | 0.049 |
| Never smokers | 338 (42.6%) | Reference | |  | Reference | |  | Reference | |  | Reference | |
| Ever smokers | 455 (57.4%) | 1.590 (1.189-2.128) |  |  | 1.342(0.996-1.809) |  |  | 1.500 (1.119-2.010) |  |  | 1.361 (1.002-1.850) |  |
| **Smoking amount (cigarettes/day)** |  |  | 0.002 |  |  | 0.104 |  |  | 0.009 |  |  | 0.035 |
| 0 | 338 (42.6%) | Reference | |  | Reference | |  | Reference | |  | Reference | |
| 1-19 | 99 (12.5%) | 1.189 (0.735-1.924) | 0.480 |  | 1.141 (0.705-1.846) | 0.593 |  | 1.167 (0.721-1.889) | 0.529 |  | 0.987 (0.591-1.649) | 0.961 |
| ≥20 | 356 (44.9%) | 1.701 (1.260-2.297) | 0.001 |  | 1.397 (1.024-1.905) | 0.035 |  | 1.589(1.174-2.151) | 0.003 |  | 1.473 (1.072-2.024) | 0.017 |
| **Smoking duration (years)** |  |  | 0.003 |  |  | 0.151 |  |  | 0.009 |  |  | 0.076 |
| 0 | 338 (42.6%) | Reference | |  | Reference |  |  | Reference | |  | Reference | |
| 1-29 | 263 (32.2%) | 1.439 (1.033-2.004) | 0.031 |  | 1.363 (0.977-1.901) | 0.069 |  | 1.356 (0.972-1.891) | 0.073 |  | 1.246 (0.878-1.770) | 0.219 |
| ≥30 | 192 (24.2%) | 1.805 (1.282-2.540) | 0.001 |  | 1.313 (0.910-1.895) | 0.146 |  | 1.704 (1.209-2.402) | 0.002 |  | 1.523 (1.059-2.191) | 0.023 |
| **Cumulative smoking consumption (pack-years)** |  |  | 0.001 |  |  | 0.129 |  |  | 0.003 |  |  | 0.012 |
| 0 | 338 (42.6%) | Reference | |  | Reference |  |  | Reference | |  | Reference | |
| 1-29 | 247 (31.2%) | 1.333 (0.943-1.884) | 0.103 |  | 1.274 (0.901-1.803) | 0.171 |  | 1.271 (0.899-1.799) | 0.075 |  | 1.110 (0.766-1.608) | 0.581 |
| ≥30 | 208 (26.1%) | 1.886 (1.358-2.619) | <0.001 |  | 1.420 (0.999-2.018) | 0.050 |  | 1.760 (1.264-2.450) | 0.001 |  | 1.649 (1.166-2.332) | 0.005 |

Abbreviations: HR: hazard ratio; CI: confidence interval. Model 1 was performed in the entire cohort, adjusted for age, T stage and N stage. Model 2 was performed in the entire cohort, excluded the age as a covariate from Model 1. Model 3 was adjusted for T stage and N stage and pre-treatment EBV DNA which performed in 673 patients.

Supplementary Table 2. Multivariable analyses of prognostic effects for smoking indicators on overall survival in male patients (Details of all variables).

| **Variable** | **OS** | | | | | |
| --- | --- | --- | --- | --- | --- | --- |
|  | **Model 1 (N=793)** | | **Model 2 (N=793)** | | **Model 3 (N=673)** | |
|  | **HR (95% CI)** | ***P* value** | **HR (95% CI)** | ***P* value** | **HR (95% CI)** | ***P* value** |
| **pre-EBV** (≥1500 vs.<1500) | — | — | — | — | 2.315 (1.699 - 3.154) | <0.001 |
| **Age** (≥45 vs. <45) | 1.759 (1.302 - 2.375) | <0.001 | — | — | — | — |
| **T stage** (T4 vs. T1-3) | 1.534 (1.161 - 2.027) | 0.003 | 1.476 (1.118 - 1.948) | 0.006 | 1.396 (1.036 - 1.880) | 0.028 |
| **N stage** (N2-3 vs. N0-1) | 1.450 (1.091 - 1.926) | 0.010 | 1.388 (1.045 - 1.843) | 0.024 | 1.215 (0.896 - 1.647) | 0.210 |
| **Smoking status (**ever smokers vs. never smokers**)** | 1.342 (0.996 - 1.809) | 0.053 | 1.500 (1.119 - 2.010) | 0.007 | 1.361 (1.002 - 1.850) | 0.049 |
| **pre-EBV** (≥1500 vs.<1500) | — | — | — | — | 2.358 (1.729 - 3.214) | <0.001 |
| **Age** (≥45 vs. <45) | 1.729 (1.277 - 2.340) | <0.001 | — | — | — | — |
| **T stage** (T4 vs. T1-3) | 1.522 (1.151 - 2.012) | 0.003 | 1.463 (1.108 - 1.932) | 0.007 | 1.374 (1.020 - 1.852) | 0.037 |
| **N stage** (N2-3 vs. N0-1) | 1.439 (1.083 - 1.913) | 0.012 | 1.378 (1.038 - 1.831) | 0.027 | 1.218 (0.899 - 1.650) | 0.204 |
| **Smoking amount (cigarettes/day)** |  | 0.104 |  | 0.009 |  | 0.035 |
| 0 | Reference | | Reference | | Reference | |
| 1-19 | 1.141 (0.705 - 1.846) | 0.593 | 1.167 (0.721 - 1.889) | 0.529 | 0.987 (0.591 - 1.649) | 0.961 |
| ≥20 | 1.397 (1.024 - 1.905) | 0.035 | 1.589 (1.174 - 2.151) | 0.003 | 1.473 (1.072 - 2.024) | 0.017 |
| **pre-EBV** (≥1500 vs.<1500) | — | — | — | — | 2.303 (1.690 - 3.137) | <0.001 |
| **Age** (≥45 vs. <45) | 1.780 (1.290 - 2.457) | <0.001 | — | — | — | — |
| **T stage** (T4 vs. T1-3) | 1.536 (1.162 - 2.030) | 0.003 | 1.472 (1.115 - 1.944) | 0.006 | 1.396 (1.037 - 1.880) | 0.028 |
| **N stage** (N2-3 vs. N0-1) | 1.451 (1.092 - 1.928) | 0.010 | 1.393 (1.049 - 1.850) | 0.022 | 1.223 (0.902 - 1.659) | 0.195 |
| **Smoking duration (years)** |  | 0.151 |  | 0.009 |  | 0.076 |
| 0 | Reference | | Reference | | Reference | |
| 1-29 | 1.363 (0.977 - 1.901) | 0.069 | 1.356 (0.972- 1.891) | 0.073 | 1.246 (0.878 - 1.770) | 0.219 |
| ≥30 | 1.313 (0.910 - 1.895) | 0.146 | 1.704 (1.209 - 2.402) | 0.002 | 1.523 (1.059 - 2.191) | 0.023 |
| **pre-EBV** (≥1500 vs.<1500) | — | — | — | — | 2.302 (1.689 - 3.136) | <0.001 |
| **Age** (≥45 vs. <45) | 1.704 (1.239 - 2.344) | 0.001 | — | — | — | — |
| **T stage** (T4 vs. T1-3) | 1.519 (1.147 - 2.011) | 0.004 | 1.449 (1.096 - 1.914) | 0.009 | 1.360 (1.008 - 1.834) | 0.044 |
| **N stage** (N2-3 vs. N0-1) | 1.448 (1.090 - 1.924) | 0.011 | 1.397 (1.052 - 1.856) | 0.021 | 1.239 (0.913 - 1.680) | 0.169 |
| **Cumulative smoking consumption (pack-years)** |  | 0.129 |  | 0.003 |  | 0.012 |
| 0 | Reference | | Reference | | Reference | |
| 1-29 | 1.274 (0.901 - 1.803) | 0.171 | 1.271 (0.899 - 1.799) | 0.175 | 1.110 (0.766 - 1.608) | 0.581 |
| ≥30 | 1.420 (0.999 - 2.018) | 0.050 | 1.760 (1.264 - 2.450) | 0.001 | 1.649 (1.166 - 2.332) | 0.005 |
