# Supplementary material for: Combination of smoking and Epstein-Barr virus DNA is a predictor of poor prognosis for nasopharyngeal carcinoma: a long-term follow-up retrospective study
Source: BMC Cancer. 2022 Dec 5;22:1262. doi: 10.1186/s12885-022-10297-w (PMC9720998; doi:10.1186/s12885-022-10297-w)
Supplement: Supplementary file 2 — Additional file 2: Supplementary Material 1. Detailed treatment regimens for the entire cohort. Supplementary Material 2. Detailed information of follow-up for the entire cohort. Supplementary Material 3. The detailed definition of the study endpoints. Supplementary Material 4. Detailed information of treatment failure for 793 male patients. Supplementary Material 5. Detailed information and results of propensity score matching (PSM) analysis. Supplementary Material 6. The results of survival analysis of only male smokers who were classified as heavy, moderate and low cigarette smoking consumers. [file 12885_2022_10297_MOESM2_ESM.docx]

**Supplementary Material 1.** Detailed treatment regimens for the entire cohort.

All patients were treated with IMRT at a total dose of 66–74 Gy for 6–8 weeks at target areas located by computed tomography (CT). In the cohort, 76 patients (7.1%) were at stage I and received IMRT treatment alone, 145 patients (13.4%) were at stage II and received concurrent chemoradiotherapy, and 629 patients (79.5%) were at stages III/IV and received concurrent chemoradiotherapy, induction chemotherapy, and/or adjuvant chemotherapy. Detailed protocols for radiotherapy and chemotherapy as follow:

(i) Radiotherapy

All patients in this study received intensity-modulated radiotherapy (IMRT) to targets located by CT. The irradiation doses were as follows. The total dose to the planning target volume (PTV) in the primary tumor area (GTVnx) was 70-74 Gy, that to the neck metastatic lymph node area (GTVnd) was 66-70 Gy, that to the high-risk clinical target volume (CTV1) was 60-62 Gy, and that to the low-risk clinical target volume (CTV2) was 50-56 Gy. The total dose was divided into 28 and 33 applications delivered once per day, 5 days per week, at a single dose of 2.12-2.24 Gy.

(ii) Chemotherapy

All chemotherapy regimens were cisplatin-based combination regimens and a complete cycle was administered every 3 weeks. The induction chemotherapy regimen (1 or 2 cycles) and adjuvant chemotherapy regimen (1-4 cycles) included TP (docetaxel 60 mg/m^2^/d or paclitaxel 135 mg/m^2^/d on day 1 and cisplatin 25 mg/m^2^/d on days 1 to 3), TPF (docetaxel 60 mg/m^2^/d or paclitaxel 135 mg/m^2^/d on day 1, cisplatin 25 mg/m^2^/d on days 1 to 3, and 5-fluorouracil 600 mg/m^2^/d on days 1 to 5), and PF (cisplatin 25 mg/m^2^/d on days 1 to 3 and 5-fluorouracil 600 mg/m^2^/d on days 1 to 5). The concurrent chemotherapy regimens (1 or 2 cycles) included cisplatin monotherapy (once every three weeks, 25 mg/m^2^/d on days 1 to 3) and TP (docetaxel 60 mg/m^2^/d or paclitaxel 135 mg/m^2^/d on day 1 and cisplatin 25 mg/m^2^/d on days 1 to 3).

**Supplementary Material 2.** Detailed information of follow-up for the entire cohort.

After therapy, all patients were routinely evaluated every 3 months within the first year, every 6 months during the second and third years, and annually thereafter. Physical examination of the head and neck, nasopharyngeal endoscopy, magnetic resonance image of the nasopharynx and neck, abdominal ultrasound, chest radiography, whole-body positron emission tomography (PET), and plasma EBV DNA measurements were performed routinely. PET/CT was further performed if necessary.

**Supplementary Material 3**. The detailed definition of the study endpoints.

The primary study endpoint was overall survival (OS), which was defined as the time between initial pathological diagnosis of NPC and all-cause death or last follow-up visit. The secondary endpoints included progression-free survival (PFS), distant metastasis-free survival (DMFS), and locoregional relapse-free survival (LRFS). PFS was defined as the time from the initial pathological diagnosis of NPC to disease progression or death from any cause, whichever occurred first, or last follow-up visit. DMFS was defined as the time from pathological diagnosis to distant metastasis detection, death, or last follow-up visit. LRFS was defined as the time from pathological diagnosis to relapse in nasopharynx or neck lymph nodes, death, or last follow-up visit.

**Supplementary Material 4.** Detailed information of treatment failure for 793 male patients.

During the median follow-up period of 66.4 months (range: 2–197 months) for male NPC patients (n = 793), a total of 317 patients (39.9%) experienced disease progression, including 67 cases of locoregional relapse (8.4%), 136 cases of distant metastasis (17.2%), 34 cases of both locoregional relapse and distant metastasis (4.3%), and 204 deaths (25.7%); 124 patients died from locoregional recurrence or distant metastasis and 80 patients died without locoregional recurrence or distant metastasis.

**Supplementary Material 5**. Detailed information and results of propensity score matching (PSM) analysis.

1. Table 1. Clinical characteristics of male patients with NPC.

| **Characteristic** | **Total male patients (N = 793)** | | | | **PSM matched male patients (N = 566)** | | |
| --- | --- | --- | --- | --- | --- | --- | --- |
|  | **Never smokers**  **(N = 338)，n (%)** | **Ever smokers**  **(N = 455)，n (%)** | ***P* value*** | **Never smokers**  **(N = 283)，n (%)** | | **Ever smokers**  **(N = 283)，n (%)** | ***P* value**** |
| **Sex** |  |  | — |  | |  | — |
| **Female** | 0 (0) | 0 (0) |  | 0 (0) | | 0 (0) |  |
| **Male** | 338 (100%) | 455 (100%) |  | 283 (100%) | | 283 (100%) |  |
| **Age (years)** |  |  | <0.001 |  | |  | 1.000 |
| **<45** | 177 (52.4%) | 160 (35.2%) |  | 133 (47.0%) | | 132 (46.6%) |  |
| **≥45** | 161 (47.6%) | 295 (64.8%) |  | 150 (53.0%) | | 151 (53.4%) |  |
| **Overall stage^b^** |  |  | 0.051 |  | |  | 0.706 |
| **I** | 26 (7.7%) | 28 (6.2%) |  | 25 (8.8%) | | 22 (7.8%) |  |
| **II** | 55 (16.3%) | 55 (12.1%) |  | 40 (14.1%) | | 39 (13.8%) |  |
| **III** | 112 (33.1%) | 133 (29.2%) |  | 86 (30.4%) | | 86 (30.4%) |  |
| **IV** | 145 (42.9%) | 239 (52.5%) |  | 132 (46.6%) | | 136 (48.1%) |  |
| **Tumor stage^b^** |  |  | 0.044 |  | |  | 0.085 |
| **T1** | 70 (20.7%) | 93 (20.4%) |  | 62 (21.9%) | | 66 (23.3%) |  |
| **T2** | 70 (20.7%) | 84 (18.5%) |  | 57 (20.1%) | | 57 (20.1%) |  |
| **T3** | 76 (22.5%) | 74 (16.3%) |  | 52 (18.4%) | | 41 (14.5%) |  |
| **T4** | 122 (36.1%) | 204 (44.8%) |  | 112 (39.6%) | | 119 (42.0%) |  |
| **Node stage^b^** |  |  | 0.052 |  | |  | 0.192 |
| **N0** | 60 (17.8%) | 64 (14.1%) |  | 50 (17.7%) | | 50 (17.7%) |  |
| **N1** | 111 (32.8%) | 125 (27.5%) |  | 84 (29.7%) | | 93 (32.9%) |  |
| **N2** | 145 (42.9%) | 220 (44.8%) |  | 130 (45.9%) | | 120 (42.4%) |  |
| **N3** | 22 (6.5%) | 46 (10.1%) |  | 19 (6.7%) | | 20 (7.1%) |  |
| **Alcohol drinking** |  |  | <0.001 |  | |  | 1.000 |
| **No** | 322 (95.3%) | 324 (71.2%) |  | 267 (94.3%) | | 267 (94.3%) |  |
| **Yes** | 16 (4.7%) | 131 (28.8%) |  | 16 (5.7%) | | 16 (5.7%) |  |
| **Family history of NPC** |  |  | 0.047 |  | |  | 1.000 |
| **No** | 309 (91.4%) | 432 (94.9%) |  | 265 (93.6) | | 264 (93.3%) |  |
| **Yes** | 29 (8.6%) | 23 (5.1%) |  | 18 (6.4%) | | 19 (6.7%) |  |

^a^ Pathologic type according to the 2005 World Health Organization (WHO) classification of tumors.

^b^ According to the 7th edition of the AJCC staging system.

*Chi-square test or Fisher’s exact test

**Cochran-Mantel-Haenszel chi-squared test

1. Figure 1 Distribution of propensity scores before and after PSM for ever smokers and never smokers. The PSM was performed by matchign on age, overall stage, tumor stage, node stage, alcohol drinking, and family history of NPC, caliper=0.05.

**
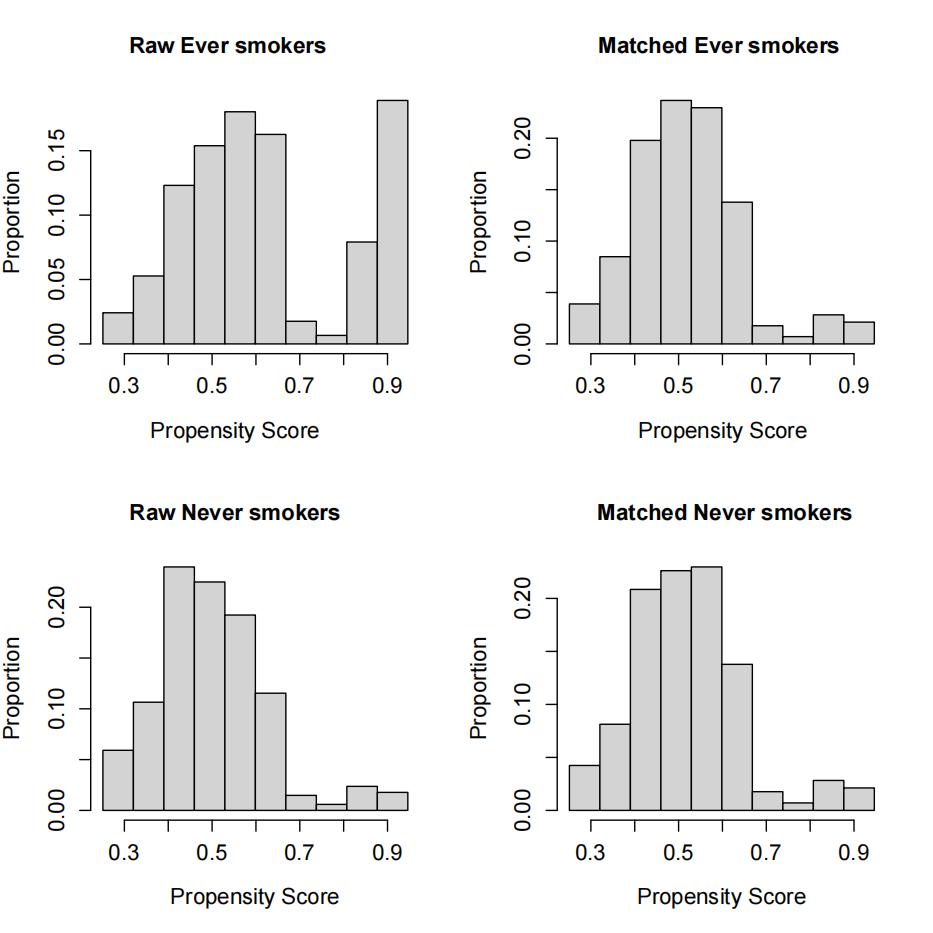
**

1. The results of survival analysis of PSM matched male patients (N = 566) who were stratified by different smoking indicators. The results showed that there were statistically significant differences between subgroups in all smoking exposure indicators (all *P* < 0.05), which can be seen in the survival curve graphs below.

Figure 2 Kaplan-Meier curves for overall survival of PSM matched male patients (N = 566) stratified by different smoking indicators: (A) smoking status. (B) smoking amount. (C) smoking duration. (D) cumulative smoking consumption.

**

**

**Supplementary Material 6**. The results of survival analysis of only male smokers who were classified as heavy, moderate and low cigarette smoking consumers.

Based on the different smoking indicators, including smoking amount, smoking duration, and cumulative smoking consumption, 455 male smokers were classified as heavy, moderate and low cigarette smoking consumers. However, no positive results was found in survival analysis (all *P* > 0.05), which can be seen in the following survival curve graphs.

Figure The Kaplan-Meier curves for overall survival of only male smokers(N = 455) classified as heavy, moderate and low cigarette smoking consumers: (A) smoking amount. (B) smoking duration. (C) cumulative smoking consumption.
